# Identification of the associations between genes and quantitative traits using entropy-based kernel density estimation

**DOI:** 10.5808/gi.22033

**Published:** 2022-06-30

**Authors:** Jaeyong Yee, Taesung Park, Mira Park

**Affiliations:** 1 Department of Physiology and Biophysics, Eulji University, Daejeon 34824, Korea; 2 Department of Statistics, Seoul National University, Seoul 08826, Korea; 3 Department of Preventive Medicine, Eulji University, Daejeon 34824, Korea

**Keywords:** genetic association, kernel density estimation, mutual information, quantitative trait

## Abstract

Genetic associations have been quantified using a number of statistical measures. Entropy-based mutual information may be one of the more direct ways of estimating the association, in the sense that it does not depend on the parametrization. For this purpose, both the entropy and conditional entropy of the phenotype distribution should be obtained. Quantitative traits, however, do not usually allow an exact evaluation of entropy. The estimation of entropy needs a probability density function, which can be approximated by kernel density estimation. We have investigated the proper sequence of procedures for combining the kernel density estimation and entropy estimation with a probability density function in order to calculate mutual information. Genotypes and their interactions were constructed to set the conditions for conditional entropy. Extensive simulation data created using three types of generating functions were analyzed using two different kernels as well as two types of multifactor dimensionality reduction and another probability density approximation method called m-spacing. The statistical power in terms of correct detection rates was compared. Using kernels was found to be most useful when the trait distributions were more complex than simple normal or gamma distributions. A full-scale genomic dataset was explored to identify associations using the 2-h oral glucose tolerance test results and γ-glutamyl transpeptidase levels as phenotypes. Clearly distinguishable single-nucleotide polymorphisms (SNPs) and interacting SNP pairs associated with these phenotypes were found and listed with empirical p-values.

## Introduction

Over the past decades, genetic association studies have been conducted to identify genetic variants associated with various traits or diseases [1–3]. Genetic susceptibility for many complex diseases is often analyzed using diagnosis-based categories, although the underlying phenotypes are usually quantitative [4,5]. A genomic association, however, does not necessarily require any classification. Therefore, the intrinsic features of an association may be better reflected by entering the quantitative distributions into the association measurement in their original form. Furthermore, some traits, such as human height, are intrinsically continuous; therefore, meaningful thresholds for categorization may not exist.

Multifactor dimensionality reduction (MDR) has been successfully used as a genomic association measurement method [6]. It can identify interacting genes, and it was originally intended for binary outcomes. This method uses the classification accuracy, measured by constructing a confusion matrix, to quantify an association. Variants of MDR have emerged. For ordered categorical traits with more than two response categories, ordinal MDR uses Kendall’s tau-b as an association measure [7]. For quantitative traits, generalized MDR (GMDR) and quantitative MDR (QMDR) have been proposed. Inheriting the original MDR, the common strategy of these methods is to classify the trait values corresponding to a genotype as a binary state. GMDR utilizes a score statistic after adjusting covariates [8]. QMDR uses T-statistics as the association measure and compares the mean values for each cell with the overall mean to classify the trait distribution [9].

Entropy-based methods of analyzing genomic associations have emerged as another stream of research [10]. According to information theory, mutual information (MI) is defined as the amount of information, or entropy, shared by two random variables [11,12]. In analyses of genomic associations, this concept can be translated into the strength of the association between the genotype and phenotype [10]. MI is regarded as a generalized correlation measure in the sense that it is not limited to linear dependence [13]. MI has been evaluated as a measure for associations and extended to machine learning [14]. The estimation of MI between discrete or categorical random variables is well established. However, when either of two variables is quantitative, estimating MI is not at all straightforward [15]. MI-based test statistics for gene-gene interactions associated with discrete trait values have been proposed [16,17]. Quantitative traits have also been considered with generalized MI, referred to as “k-way interaction information,” but with the assumption of a normal distribution [18]. A more direct estimation of MI with quantitative traits has been suggested using the m-spacing entropy measure [19]. This method estimates MI utilizing the observed spacing of order m between quantitative trait values, without any assumption or classification attempt. The probability density tends to be inversely proportional to the spacing between data points. M-spacing elaborates upon this notion by considering the spacings beyond the immediately adjacent points, resulting in more accurate estimations of probability density. This is the basis, in turn, for a more precise determination of entropy and MI.

Here, we propose another way of analyzing genomic associations for quantitative traits based on the kernel density estimation (KDE). KDE estimates a distribution function by summing kernels over the domain, or the observed data points. Kernels are designed to be normalized and non-negative functions, symmetric around each data point [20]. MI would be obtained with these estimated distribution functions of quantitative traits. We examined the KDE method by varying the kernels and using adaptive bandwidth for them to determine the most proper way of combining KDE and MI estimations for genomic association data. Associations with gene-gene interactions were investigated with quantitative traits of simulation and real datasets. Statistical power was analyzed in terms of the correct detection rates for extensive sets of simulation data obtained by KDE, two types of MDR, and m-spacing. This comparison showed that using kernels may be more useful than other methods when the trait distributions are more complex than simple normal or gamma distributions. A full-scale genomic dataset with the phenotype of the 2-h oral glucose tolerance test was selected from the Korean Association Resource (KARE) project [21], because the distributions were found to be complex. Additionally, γ-glutamyl transpeptidase (γ-GTP) levels were explored as a phenotype. Single-nucleotide polymorphisms (SNPs) and interacting SNP pairs associated with this phenotype were clearly identified and listed with empirical p-values.

## Methods

**Fig. 1:**
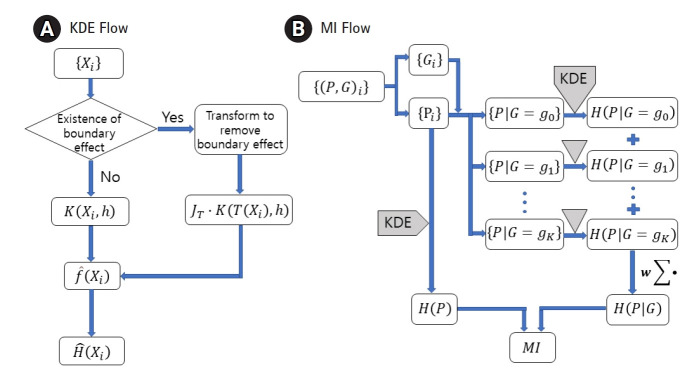
Flow charts for the kernel density estimation (KDE) (A) and the mutual information (MI) (B). Entropy, H, can be estimated from a dataset, {Xi}, sampled from a distribution of density f. Transformed kernel should be used when the boundary effect is not negligible. MI can be obtained by applying KDE to phenotype (P) and genotype (G) data and then combining the results.

MI between the genotype and the quantitative phenotype is investigated to establish a genomic association. Measuring MI requires estimating the entropy and conditional entropy. To estimate them for a quantitative trait, the probability density function (pdf) needs to be estimated first. KDE has been adopted to estimate the pdf for distributions with or without a boundary effect. Fig. 1A shows the use of an additional factor *J_T_* with the kernel when the variable is transformed. Fig. 1B visualizes when to apply KDE to genomic data to obtain MI.

### Definition of entropy and MI

When the probability density function, *f*(*x*)**,** is known, the entropy, *H*, is defined in the integral form of the pdf as below, which is also called the differential entropy [22].


(1) H (f)=-∫f (x) ln f (x) dx

MI is defined as the difference between the entropy of one set and that conditioned by the other set, where two sets are interchangeable. MI can quantify the association between two sets [11], which, in the scope of this paper, would be paired observations of the phenotype and genotype values. MI is obtained by the difference between the two entropies above.


(2) MI=H (P)-H (P∣G)

, where *H*(*P*) is the amount of information contained in the phenotype distribution [10]. The conditional entropy *H*(*P*|*G*) measures the amount of information still necessary to describe the phenotype distribution when the genotype is known. Equivalently, it is the amount of information that the phenotype distribution does not share with the genotype. Therefore, MI in Eq. (2) quantifies the amount of information that the phenotype and genotype distributions share. The more information they share, the more strongly the genetic information contributes to the phenotype.

### Entropy by KDE

To estimate the entropy in (1), we first need to estimate *f*(*x*) from the data [22]. Let {*X_i_*} denote the set of random samples drawn from a distribution with density *f*. Then, the entropy *H* can be estimated as follows.


(3) $$\hat{H}=-\frac{1}{n}\sum_{i=1}^{n}\text{ln }\hat{f}\;\left(X_{i}\right)$$

The estimation of entropy now becomes equivalent to the estimation of *f* (i.e., a pdf). For that purpose, KDE can be used to estimate *f*. A simple and known function *K*, called a kernel, may be defined around each data point and summed for the estimation of a pdf, as shown below [23].


(4) $$\hat{f}\;(x)=\frac{1}{n}\sum_{j=1}^{n}\frac{1}{h}K\;\left(\frac{x-X_{j}}{h}\right)$$

Here *K*(*u*) should be non-negative and symmetric for our purpose, while satisfying the normalization condition. The requirement for a kernel function that it should be normalized in the range of its argument also ensures the normalization of the pdf [20]. It should be noted that at an arbitrary point *x*, the pdf is determined by the sum of *n* individual kernel functions whose centers are at *x* = *X_j_*. The width of a kernel function is controlled by the bandwidth *h*.

### Kernels for a distribution with a boundary

**Fig. 2:**
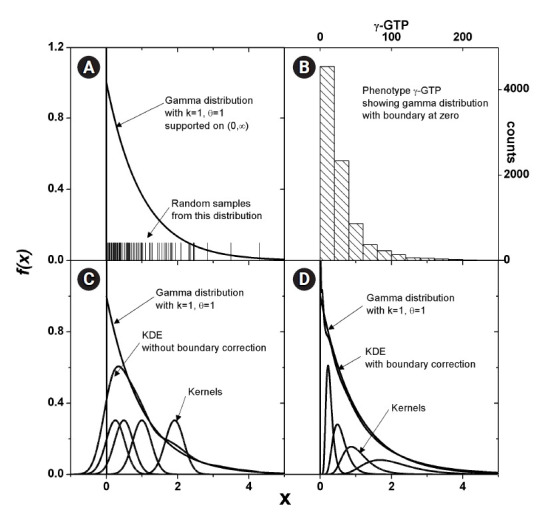
Estimation of density for the distributions with boundary. Gamma distribution, which has boundary at 0, is shown (A). Histogram of γ-glutamyl transpeptidase (γ-GTP) that follows such distributions is plotted (B). Kernels in x-space would always estimate a tailed density outside the boundary as shown (C), which should lead to the estimation of entropy for the more diffused distribution. Corrected density (D) fits better without crossing the boundary.

Some phenotype distributions have distinct boundaries. For example, let us examine the phenotype of γ-GTP levels, as shown in Fig. 2B. Unlike usual Gaussian distributions, which can be found with weight or blood pressure measurements, this histogram is crowded near the boundary value of zero. A skewed distribution like this can be modeled with a gamma distribution, as presented in Fig. 2A. The range supported is (0, ∞). As suggested in Eq. (4), KDE estimates the pdf as the sum of kernels, which is symmetric around each data point. In Fig. 2C, a few kernels are shown along with the estimated pdf. When the density value is significant near the boundary, as in this case, the estimated pdf inevitably has tails outside the supported range. The normality of the pdf is then broken, and the estimated shape of the pdf may not reach the real distribution. This eventually results in an inaccurate estimation of MI. One remedy for this is to use a kernel in the following form [24].


(5) $$\hat{f}\;(x)=\frac{1}{n}\sum_{j=1}^{n}\frac{1}{xh}\;K\;\left(\frac{\text{ln }x-\text{ln }X_{j}}{h}\right)$$

Here, the kernel is symmetric in ln *x* space, whose range is (−∞, ∞). The different Jacobian between Eqs. (4) and (5) should be noted; this can be obtained straightforwardly from the fact that the normality of the kernel function is defined as below.


(6) $$\int_{-\infty }^{\infty }K\;(u)du=1$$

Transforming back to *x* space, the estimated pdf fits better, as shown in Fig. 2D. The kernels will not be symmetric in *x* space, and the shape will be dependent on the data point *X_j_*, around which the kernels are estimated.

### Choice of the kernel function

Several types of kernel functions have been proposed that satisfy the symmetric and non-negative conditions imposed for our purpose [23]. Among them, the Epanechnikov kernel has the highest efficiency, which means that it has the smallest asymptotic mean integrated squared error over other kernels when the number of data points is the same [23]. It has a parabolic form as below.


(7) $$K(u)=\frac{3}{4}\left(1-u^{2}\right)1\left(\left|u\right|\leq 1\right)$$

The indicator function, 1(..), is used. Meanwhile, the Gaussian kernel has about 5% lower efficiency, which means it requires 5% more data points to achieve the same error level as the Epanechnikov kernel. However, the Gaussian kernel is widely used because of its mathematical convenience. It has the form given below.


(8) $$K(u)=\frac{1}{\sqrt{2\pi }}\text{exp}\left(-\frac{u^{2}}{2}\right)$$

The Epanechnikov kernel is also more advantageous for computation due to its relative simplicity, which becomes an important factor with genomic data containing an extensive set of genotypes [25]. We examined these two kernels.

### Determination of bandwidth

As can be seen in Eqs. (4) and (5), the bandwidth *h* should be determined to make arguments for kernel functions. It also plays the role of a weight factor for the sum of kernels at each point. The value of bandwidth can be deduced by setting the derivative of the asymptotic mean integrated squared error with respect to the bandwidth as zero [26]. However, it has a differentiation term of the pdf, which is obviously unknown. An acknowledged replacement is the sample standard deviation, *σ̂*, and a constant specific to the kernel used [26]. Its expression is as follows, where *n* is the number of data points.


(9) $$h=\hat{\sigma }C_{\nu }\;(K)\;n^{-1/(2\nu +1)}$$

The bandwidth in Eq. (9) now depends on the shape of data distribution and the kernel shape. We used *ν* = 2 and *C*_2_=2.34, 1.06 for the kernels in Eqs. (7) and (8), respectively.

### MI by entropy and conditional entropy

Combining Eq. (3) with Eqs. (4) or (5), the entropy for the whole phenotype, *P*, can be estimated as follows.


(10) $$H\;(P)=[\begin{array}{c} -\frac{1}{n}\sum_{i=1}^{n}\text{ln }\left[\frac{1}{n}\sum_{j=1}^{n}\frac{1}{b}K\;\left(\frac{X_{i}-X_{j}}{h}\right)\right],\;\text{no boundary effect}\\ -\frac{1}{n}\sum_{i=1}^{n}\text{ln }\left[\frac{1}{n}\sum_{j=1}^{n}\frac{1}{X_{i}h}K\;\left(\frac{\text{ln }X_{i}-\text{ln }X_{j}}{h}\right)\right],\;\text{boundary effect} \end{array}$$

For computing the conditional entropy, the phenotype set needs to be divided according to the corresponding genotypes, represented as {*P*|*G*=*g*}. Let *g* indicate each genotype and *d* be used for the order of genomic interaction. Because each SNP has three different forms (AA, Aa, and aa), *d*-order interacting SNPs should have 3*^d^* possible genotypes. The conditional entropy can now be obtained by summing the above KDE calculations on each subset, weighted by the subset size, as below.


(11) $$\begin{array}{l} H\;(P|G)=\sum_{g=0}^{3^{d}-1}prob(G=g)H\;\left(P|G=g\right)\\ =[\begin{array}{c} -\sum_{i=1}^{n}\frac{1}{n}\;\left[\sum_{i=1}^{n_{g}}\text{ln }\left[\frac{1}{n_{g}}\sum_{j=1}^{n_{g}}\frac{1}{h^{g}}K\;\left(\frac{X_{i}^{g}-X_{j}^{g}}{h^{g}}\right)\right]\right],\;\text{no boundary effect}\\ -\sum_{g=0}^{3^{d}-1}\frac{1}{n}\;\left[\sum_{i=1}^{n_{g}}\text{ln }\left[\frac{1}{n_{g}}\sum_{j=1}^{n_{g}}\frac{1}{X_{i}^{g}h^{g}}K\;\left(\frac{\text{ln }X_{i}^{g}-\text{ln }X_{j}^{g}}{h^{g}}\right)\right]\right],\;\text{boundary effect} \end{array} \end{array}$$

We denote the *i*-th phenotype data in subset {*P*|*G*=g} by $X_{i}^{g}$. The bandwidths are also defined independently within each subset. Finally, MI is obtained by the difference between the two entropy values above, as noted in Eq. (2).

### Estimation of p-values

If statistical significance of the obtained MI is required, the p-value is estimated by random permutation of the trait values among samples to make the resultant dataset satisfy the null hypothesis. The maximum MI value of all genotype combinations from this dataset would form a single point of the null distribution of MI constructed by repeated random permutations. Counting the number of points in this null distribution that are larger than or equal to the observed MI would give the desired empirical p-value.

## Results

### Generation of simulation data

The application of KDE to a genomic association study and its performance were examined with a simulated dataset. Simulated data were generated based on the Velez models [27], which assume 2-order SNP interactions for binary phenotypes. Penetrance values, *t_ij_*, were tabulated for each of the nine possible genotype combinations of two interacting SNPs, along with specified values of the minor allele frequency (MAF) and heritability. To generate quantitative values, we took the penetrance as the mean of the distribution from which the trait value was sampled. Three types of distributions were considered. The first type was a normal distribution, as given below.


(12) $$y_{ij}\sim N\;\left(t_{ij},\sigma^{2}\right)\;\;\;i,j=0,1,2\;or\;AA,Aa,aa$$

Another was a gamma distribution, shown below.


(13) $$y_{ij}\sim \Gamma \;(k,\theta )=\Gamma \;\left(\frac{t_{ij}^{2}}{\sigma^{2}},\frac{\sigma^{2}}{t_{ij}}\right)\;\;\;i,j=0,1,2\;\text{or }AA,Aa,aa$$

It should be noted how the penetrance, *t_ij_*, was used in the distribution functions above, while σ remained a free parameter. When the penetrance, *t_ij_*, was larger or smaller than the overall average value, the class of the samples for the genotype *ij* was assigned as high or low risk, respectively. To simulate various situations, three distinct values of σ, (0.8, 1.0, 1.2), were assigned for high- and low-risk subgroups, independently establishing nine different cases. To further investigate the trait distribution, a third type of trait value sampling was done from a mixed form as shown below, with α set to 0.2.


(14) $$y_{ij}\sim [\begin{array}{l} \left(N\;\left(t_{ij}-\alpha ,\sigma^{2}\right)+N\;\left(t_{ij}+\alpha ,\sigma^{2}\right)\right)/2\\ N\;\left(t_{ij},\sigma^{2}\right) \end{array}\;\;\;\text{for }[\begin{array}{l} \text{high}\\ \text{low} \end{array}\;\text{risk}$$

The high-risk term in Eq. (14) should not be confused with the sum of normally distributed random variables. In that case, it would make just another normal distribution. Here, the high-risk term was intended to be a Gaussian mixture distribution with double peaks. With Eq. (14), the trait value was generated from a bimodal distribution if *t_ij_* was found to be larger than the overall average (i.e., a high-risk case). There were also nine combinations of σ. The number of SNPs was taken as 20 with a single causal pair and 400 samples. The Velez model has seven heritability values, each of which has five different penetrance tables for two different MAFs. For each of those 70 models, along with nine σ combinations, 100 simulations were conducted, yielding 70×90×100 files for the three distribution schemes, respectively. In all, for each of the seven heritability values, simulated datasets generated from 10 models (five penetrance tables and two MAFs), with nine variations in high- and low-risk samplings from the three types of distributions were considered.

### Demonstration of MI

**Fig. 3:**
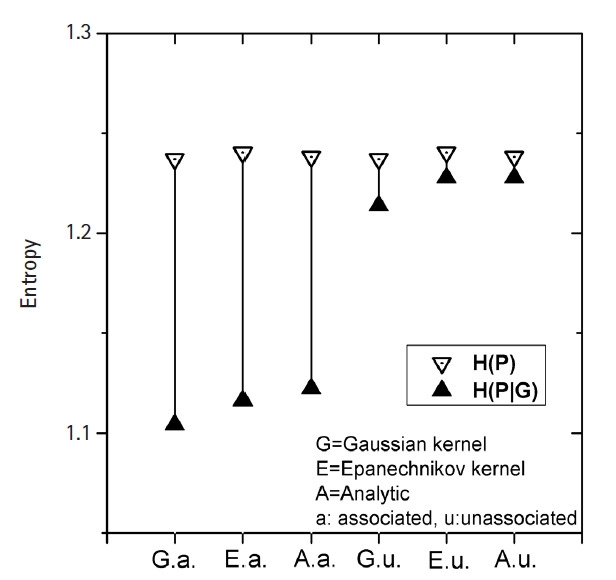
Demonstration of the association strength of a simulated genomic data obtained by kernel density estimation. Length of the vertical line between the paired points of H(P) and H(P|G) represents the association strength measure by mutual information.

Fig. 3 shows how MI works for genomic data. The simplest form of simulation data following Eq. (12), with large heritability (0.4), a MAF of 0.2, and a fixed σ of 1.0 was used. The leftmost three vertical lines were for the intended causal SNP pair that was simulated as having strong association. The rightmost three lines were for an arbitrarily chosen SNP pair that was supposed to have little association. KDE was performed on these two SNP pairs with Epanechnikov and Gaussian kernels. Analytic calculations for MI were also conducted, taking advantage of the fact that the analytic form of entropy for a normal distribution was given as $\text{ln }\left(\sigma \sqrt{2\pi e}\right)$. MI values were represented by the length of the vertical bar connecting *H*(*P*) and *H*(*P*|*G*) values, as defined in Eq. (2). Compared to the unassociated MI, the MI for the associated pair was found to be quite large. Their distinction was clear. The Epanechnikov kernel yielded a closer MI to the analytic result, which should be very close to the true value, than the Gaussian kernel.

### Comparison of hit ratios

**Fig. 4:**
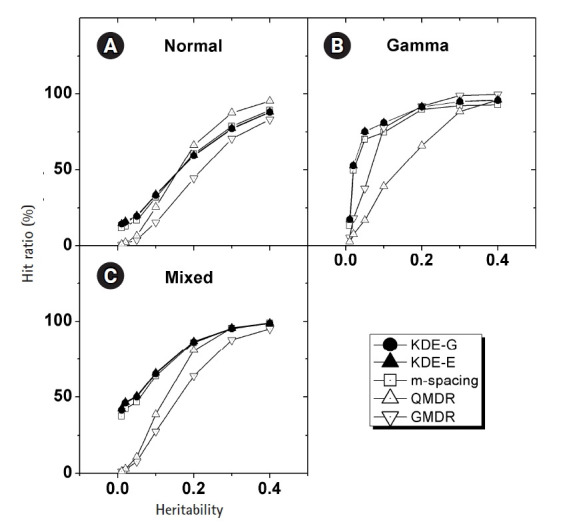
(A–C) Comparison of the hit ratios. Correct detection rates of the causal pair are compared with other method with respect to the heritability. Plots are separated by the generation schemes of the simulation data examined. KDE, kernel density estimation; QMDR, quantitative multifactor dimensionality reduction; GMDR, generalized multifactor dimensionality reduction.

In Fig. 4, the empirical power of our KDE method to identify the causal pair was investigated with the simulation data. The hit ratios using the Epanechnikov (KDE-E) and Gaussian (KDE-G) kernels were compared with the results from other methods (m-spacing, QMDR, and GMDR). Each point in the plot with respect to heritability was obtained from calculations of the hit ratio, taking all of the simulation conditions into consideration. Datasets from the normal and mixed generation functions were analyzed using the “no boundary effect” options in Eqs. (10) and (11), while those from the gamma generation function were analyzed as having a boundary effect. The results are plotted separately in (A)–(C). The two kernels showed quite similar performances throughout all the conditions. Considering the simplicity of the mathematical form, therefore, the Epanechnikov kernel should be chosen whenever the amount of calculation is heavy. For the normal and mixed cases in Fig. 4A and 4C, m-spacing results overlapped with the KDE results in high-heritability regions, although small discrepancies might exist for low-heritability regions. However, GMDR and QMDR showed somewhat different performances. In these two cases, shown in Fig. 4A and 4C, only QMDR for high-heritability regions with a normal distribution outperformed KDE, while GMDR showed the lowest performance regardless of the conditions. In Fig. 4B, for the phenotypes whose values were drawn from gamma distributions, KDE outperformed all other methods, regardless of the choice of kernels. GMDR performed best only in the two highest-heritability regions. QMDR and GMDR showed an obvious pattern of performance reversal depending on the data generation schemes. GMDR uses a scoring system, and m-spacing does not assume any pdf shape. Therefore, their performance depends little on the shape of the distributions. In contrast, QMDR tries dichotomization, which may take more advantage of symmetric than asymmetric distributions, such as gamma distribution. This may explain the performance reversal between them.

KDE is also a non-parametric method, as is m-spacing. With a symmetric distributions in Fig. 4A and 4C, KDE’s performance was found to overlap with that of m-spacing, while showing slightly better performance in the low-heritability region. With a heavily skewed distribution, as in Fig. 4B, KDE showed consistently better performance, although not substantially so, throughout the heritability regions. A gamma distribution simulation was designed such that the shape should be distinct from the normal case, with the choice of σ in Eq. (13). Since *t_ij_* in that equation is penetrance, which should be smaller than 1, the resultant gamma distribution would have a shape parameter, *k*, smaller than the scale parameter, θ, in most cases because of the used σ values. This condition results in a quite skewed gamma distribution, as intended, giving rise to the boundary effect. KDE, as designed, showed consistency and better performance than m-spacing, QMDR, and GMDR, with the exception mentioned above, regardless of the distribution shapes.

### Type I error rate

To examine the type I error rate, the same process used to build the simulation dataset was adopted to construct the null dataset, except that no causal pairs were intended. With the null dataset, the empirical p-value was evaluated by permuting the phenotype part 1,000 times. The p-value evaluation was repeated with the entire null dataset. Counting the number of instances in which the p-value obtained turned out to be smaller than the significance level, which was taken as 0.05, indicates the type I error. Table 1 presents the results. For heritability variation, a total of 9,000 (9 high-low risk deviation combinations × 1,000 repetitions) p-values were produced to estimate the type I error rate for each cell in this table, while for the MAF cells, 31,500 (7 heritability values × 5 penetrance values × 1,000 repetitions) p-values were used. The Epanechnikov kernel was employed. The estimated type I error rate was close to 5% (range, 4.6% to 5.8%), as would be expected if our method preserved this rate. The preservation of type I error by our method was verified over MAF and heritability conditions regardless of the shape of the functions for generating the simulation data.

### Application to real data 2-hour oral glucose tolerance test

**Fig. 5:**
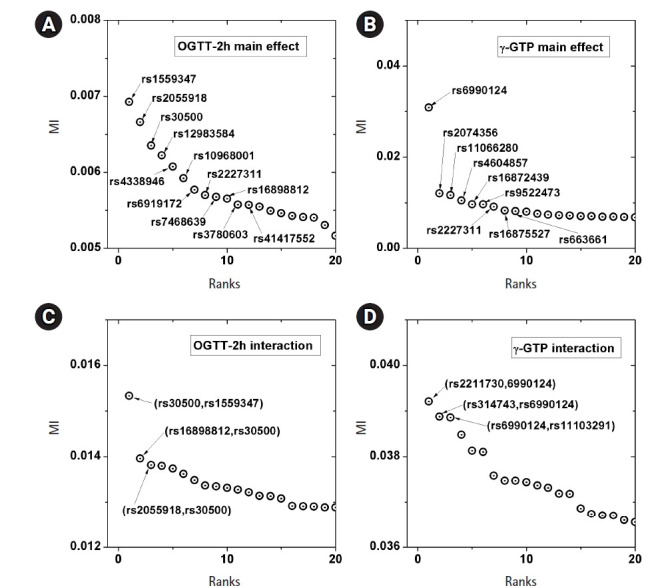
Scree plots of the associations. Top associated main effects of a single nucleotide polymorphism (SNP) (A, B) and 2-order interacting SNPs (C, D), for the phenotypes of 2-hour oral glucose tolerance test (OGTT-2h) and γ-glutamyl transpeptidase (γ-GTP), respectively. MI, mutual information.

A genome-wide dataset from the KARE project [21] was investigated for the phenotype of 2-hour oral glucose tolerance test (OGTT-2h) results, as well as γ-GTP levels. The dataset comprised 8,387 valid samples genotyped for 327,872 SNPs over 22 chromosomes. OGTT-2h is often used to diagnose diabetes, with two critical values (140 and 200 mg/dL) [28], as tabulated in Table 2. The OGTT-2h distribution was not too skewed to be regarded as the gamma distribution examined in this paper. Because of the three-stage diagnosis due to the two critical values, a more elaborate categorization than high and low risk might be necessary. Therefore, the OGTT-2h distribution may be explained better with a more complex distribution than a simple normal distribution. Instead, the mixed form examined in Fig. 4C may be appropriate for OGTT-2h. Fig. 5 shows the scree plots for the association strengths estimated with Epanechnikov kernels for the main effect (A) and two-order interactions (B). The top SNPs were identified by rs numbers. The distinction can be observed very clearly, especially in Fig. 5C. In Table 2, the details of the identified SNPs are listed. Among them, rs30500 was found to have a major association with type 1 diabetes by a previous report [29], while rs3780603 was also listed as having an association with type 2 diabetes in another study [30]. It has been suggested that glucose levels can be a prognostic factor in ovarian carcinoma [31]. Interestingly, rs2227311 in Table 3 was also listed as being associated with the risk of ovarian cancer [32]. Diabetic ketoacidosis was recently reported to have an effect on pulmonary disease [33], and rs41417552, found to be associated with the OGTT-2h phenotype, was also reported to be one of the seven associated SNPs associated with pulmonary edema [34]. The top two-order interaction effects on OGTT-2h are listed in Table 3. rs30500, which was selected by the main effect, was also found to participate in the interaction. Its interaction with rs1559347 distinguished itself quite prominently in the two-order association with OGTT-2h.

### Application to real data (γ-GTP)

The γ-GTP distribution was found to be skewed enough to be regarded as the gamma distribution. Therefore, an analysis was performed by KDE with the boundary effect considered. Fig. 5 shows the scree plots for the association strengths estimated with Epanechnikov kernels for the main effect (B) and two-order interactions (D). A clear distinction can be observed, especially in Fig. 5B. Table 4 lists the details of the identified SNPs. The newly found rs6990123 showed an outstanding association strength compared to others, and it participated in two-order interactions, as shown in Table 5, to make top associated pairs with SNPs absent from the list of the top main effects. rs2074356 was reported to have a strong association with γ-GTP levels [35], and rs11066280 was reported to have a strong association with type 2 diabetes, which is closely related to γ-GTP [36]. rs12229654, which has been reported to be associated with both γ-GTP and high-density lipoprotein cholesterol [37], was also found.

## Discussion

We investigated genomic associations with quantitative traits, including genomic interactions. Entropy-based MI can measure the association strength if the entropy of the trait could be estimated both by itself and as conditioned on the genotypes. We estimated entropy through KDE.

We explored and compared two types of kernel functions for KDE. The Epanechnikov kernel involves a far lower computational burden than the Gaussian kernel, but it was found to be as powerful as the Gaussian kernel for the genomic association task. There are several other kernels whose efficiencies lie between the Epanechnikov and Gaussian kernels, but under the non-negativity and symmetry constraint, their shapes are quite similar, especially in that their extents are limited by the indicator function, unlike the Gaussian kernel. Therefore, the two kernels investigated may lie at two extremes in terms of efficiency and how they are defined. Other kernels are expected to provide similar results.

When the dataset is made from a skewed distribution with a crowded boundary, using a symmetric kernel inherently leads to an extended tail outside the supported range. A consequence is an incorrect estimation of the association. The real data for γ-GTP, which we reported in the present analysis, may not be correctly analyzed with a usual symmetric kernel. We suggested defining a transformed argument in the kernel to confine the sum of the kernel functions within the supported range. Through these tactics, the hit ratios were found to be stable and superior to those from other methods.

The proposed method can be extended to multivariate phenotype traits, while m-spacing is intrinsically a univariate method. Multivariate traits should be the natural extension of this paper. When the real data are expected to be more complex, beyond a dichotomous classification, our method in this paper would therefore be a legitimate candidate. Phenotypes with more than one threshold can be found, one of which is the OGTT-2h phenotype analyzed here. Simultaneous associations of SNPs were found with the phenotypes that have been suggested to have OGTT-2h-related traits as a prognostic factor. Therefore, these SNP findings may provide additional evidence for the reported pathways. This might be a benefit of analyzing quantitative traits in their original form.

## Figures and Tables

**Table 1:** Type I error estimation with a significance level (*α*) of 0.05

Type I error rate (%)	Normal	Gamma	Mixed
MAF			
0.4	5.0	5.3	4.9
0.2	4.7	5.7	5.0
Heritability			
0.4	4.8	4.8	5.3
0.3	4.9	4.9	4.6
0.2	4.6	5.6	5.0
0.1	4.7	5.0	5.1
0.05	5.0	5.7	4.9
0.02	4.7	5.8	5.2
0.01	5.0	5.8	4.6
Overall	4.8	5.4	5.0

MAF, minor allele frequency.

**Table 2:** Main effect found by KDE for OGTT-2h with KARE samples

Rs ID	Chromosome	MI	p-value	Reference
rs1559347	16	0.0069	2 × 10^−5^	-
rs2055918	4	0.0066	3 × 10^−5^	-
rs30500	5	0.0064	4 × 10^−5^	[29]
rs12983584	19	0.0062	4 × 10^−5^	-
rs4338946	2	0.0061	4 × 10^−5^	-
rs10968001	9	0.0059	4 × 10^−5^	-
rs6919172	6	0.0058	4 × 10^−5^	-
rs2227311	13	0.0057	4 × 10^−5^	[32,33]
rs7468639	9	0.0057	4 × 10^−5^	-
rs16898812	5	0.0057	4 × 10^−5^	-
rs3780603	9	0.0055	4 × 10^−5^	[30]
rs41417552	5	0.0055	4 × 10^−5^	[34]

KDE, kernel density estimation; OGTT-2h, 2-hour oral glucose tolerance test; KARE, Korean Association Resource; MI, mutual information.

**Table 3:** Interactions found by KDE for OGTT-2h with KARE samples

Rs ID pair	Chromosome	MI	p-value
(rs30500, rs1559347)	(5,16)	0.0153	1 × 10^−5^
(rs16898812, rs30500)	(5,5)	0.0140	1 × 10^−5^
(rs2055918, rs30500)	(4,5)	0.0138	1 × 10^−5^

KDE, kernel density estimation; OGTT-2h, 2-hour oral glucose tolerance test; KARE, Korean Association Resource; MI, mutual information.

**Table 4:** Main effect found by KDE for γ-GTP with KARE samples

Rs ID	Chromosome	MI	p-value	Reference
rs6990124	8	0.0309	1 × 10^−5^	-
rs2074356	12	0.0120	3 × 10^−5^	[35]
rs11066280	12	0.0117	4 × 10^−5^	[36]
rs4604857	11	0.0105	1.1 × 10^−4^	-
rs16872439	8	0.0097	2.3 × 10^−4^	-
rs9522473	13	0.0096	2.4 × 10^−4^	-
rs2227311	13	0.0091	3.7 × 10^−4^	-
rs16875527	4	0.0083	7.8 × 10^−4^	-
rs663661	10	0.0081	8.7 × 10^−4^	-
rs398182	22	0.0080	9.5 × 10^−4^	-
rs12229654	12	0.0075	1.42 × 10^−3^	[37]

KDE, kernel density estimation; γ-GTP, γ-glutamyl transpeptidase; KARE, Korean Association Resource; MI, mutual information.

**Table 5:** Interactions found by KDE for γ-GTP with KARE samples

rs ID pair	Chromosome	MI	p-value
(rs2211730, rs6990124)	(8,8)	0.0392	1 × 10^−5^
(rs314743, rs6990124)	(5,8)	0.0389	1 × 10^−5^
(rs6990124, rs11103291)	(8,9)	0.0389	1 × 10^−5^

KDE, kernel density estimation; γ-GTP, γ-glutamyl transpeptidase; KARE, Korean Association Resource; MI, mutual information.
